# Cardiovascular and hepatic disease associations by magnetic resonance imaging: A retrospective cohort study

**DOI:** 10.3389/fcvm.2022.1009474

**Published:** 2022-10-17

**Authors:** Alan C. Kwan, Nancy Sun, Matthew Driver, Patrick Botting, Jesse Navarrette, David Ouyang, Shehnaz K. Hussain, Mazen Noureddin, Debiao Li, Joseph E. Ebinger, Daniel S. Berman, Susan Cheng

**Affiliations:** ^1^Departments of Cardiology, Internal Medicine, Biomedical Sciences, and Imaging, Smidt Heart Institute and Biomedical Imaging Research Institute, Cedars Sinai Medical Center, Los Angeles, CA, United States; ^2^Department of Public Health Sciences, School of Medicine and Comprehensive Cancer Center, University of California, Davis, CA, United States

**Keywords:** cardiac MRI, hepatic fibrosis, liver disease, NAFLD, FIB-4

## Abstract

**Background:**

Hepatic disease is linked to cardiovascular events but the independent association between hepatic and cardiovascular disease remains unclear, given shared risk factors.

**Methods:**

This was a retrospective study of consecutive patients with a clinical cardiac MRI (CMR) and a serological marker of hepatic fibrosis, the FIB-4 score, within one year of clinical imaging. We assessed the relations between FIB-4 scores grouped based on prior literature: low (< 1.3), moderate (1.3–3.25), and high (>3.25), and abnormalities detected by comprehensive CMR grouped into 4 domains: cardiac structure (end diastolic volumes, atrial dimensions, wall thickness); cardiac function (ejection fractions, wall motion abnormalities, cardiac output); vascular structure (ascending aortic and pulmonary arterial sizes); and cardiac composition (late gadolinium enhancement, T1 and T2 times). We used Poisson regression to examine the association between the conventionally defined FIB-4 category (low <1.3, moderate 1.3–3.25, and high >3.25) and any CMR abnormality while adjusting for demographics and traditional cardiovascular risk factors.

**Results:**

Of the 1668 patients studied (mean age: 55.971 ± 7.28, 901 [54%] male), 85.9% had ≥1 cardiac abnormality with increasing prevalence seen within the low (82.0%) to moderate (88.8%) to high (92.3%) FIB-4 categories. Multivariable analyses demonstrated the presence of any cardiac abnormality was significantly associated with having a high-range FIB-4 (prevalence ratio 1.07, 95% CI: 1.01–1.13); notably, the presence of functional cardiac abnormalities were associated with being in the high FIB-4 range (1.41, 1.21–1.65) and any vascular abnormalities with being in the moderate FIB-4 range (1.22, 1.01–1.47).

**Conclusions:**

Elevated FIB-4 was associated with cardiac functional and vascular abnormalities even after adjustment for shared risk factors in a cohort of patients with clinically referred CMR. These CMR findings indicate that cardiovascular abnormalities exist in the presence of subclinical hepatic fibrosis, irrespective of shared risk factors, underscoring the need for further studies of the heart-liver axis.

## Introduction

Increased cardiovascular morbidity is seen in patients across the spectrum of liver disease, including conditions such as cirrhotic cardiomyopathy in advanced liver disease, and accelerated atherosclerosis and arrhythmia in earlier stages of liver disease (1–3). Despite multiple postulated links, mechanisms underlying the progression from subclinical to overt cardiovascular disease in the setting of liver pathology remain uncertain. Furthermore, common risk factors that are shared between increasingly prevalent hepatic conditions, such as non-alcoholic fatty liver disease (NAFLD), and cardiovascular disease can obscure the potentially causal relationships between the two disease entities.

An improved understanding of the consistently observed associations between liver and cardiac disease can be achieved by leveraging sensitive and specific phenotyping methods applied in at-risk populations. Cardiac magnetic resonance imaging (CMR) can identify subtle changes in cardiac structure and function, vascular size and structure, and myocardial composition (4). Additionally, basic serological screening for hepatic disease using the FIB-4 score has been used to assess and classify the degree of hepatic fibrosis (5, 6). Therefore, in a real-world patient population-based cohort, we used these diagnostic methods to investigate the relation between subclinical hepatic and cardiovascular disease while adjusting for shared cardiometabolic risk factors. By examining the association of abnormalities seen on CMR with graded measures of hepatic fibrosis, we sought to elucidate the connection between hepatic and cardiovascular disease to shed light on directions for potential future targeted therapies to prevent cardiovascular morbidity within patients with hepatic disease.

## Methods

### Study sample

We assessed all patients who received a clinically-referred CMR from 1/1/2010 to 4/1/2021 within the Cedars Sinai Health System (CSHS), a large quaternary care system which serves a diverse catchment area of over 2 million individuals with 1.66 million registered outpatients in the major metropolis of Los Angeles, California. An automated electronic medical record (EMR) data pull using structured queries by clinical informatics tools (DBeaver Enterprise Database Manager v22.0.0, Python v3.9.0) was used to obtain demographic and clinical characteristics and the laboratory values comprising the FIB-4 score, including age, platelet count, aspartate transaminase, and alanine transaminase levels within one year prior to CMR. A sequential subgroup of 20 patients were manually assessed to ensure that lab values and characteristics reflected in the data pull were accurately measured, with no evidence of discrepancy. We excluded patients with insufficient data to generate the FIB-4 score within the year prior to CMR. The study was IRB approved, and a waiver of informed consent was provided for this retrospective study.

### Cardiac MRI assessment

CMR results were drawn *post-hoc* from a prospective cohort of including all clinical patients receiving CMR within CSHS which contained structured measurements recorded during initial clinical read. The measurements were organized into four distinct domains with multiple sub-components. The domains included cardiac structure with sub-components of left and right ventricular end diastolic volumes, left and right atrial 4-chamber dimensions, and septal and lateral wall thickness from steady-state free precession (SSFP, standard non-contrast views of cardiac structure and function) cine images; cardiac function including left and right ventricular ejection fraction, presence of wall motion abnormalities, and left ventricular cardiac output from SSFP cine images; vascular structure consisting of ascending aortic and pulmonary arterial sizes measured on axial Half-Fourier Acquisition Single-shot Turbo spin Echo imaging (HASTE, standard axial thoracic T2-weighted views) images; and cardiac composition which included late gadolinium enhancement (LGE, a marker of cardiac injury and scarring) on gradient echo magnitude and phase sensitive inversion recovery images, T1 times from modified Look-Locker inversion recovery sequences, and T2 times from T2-prepped SSFP sequences. Each sub-component was assessed as within or outside of normal range, as provided by Kawel-Boehm et al. (7) (Table 1).

**Table 1:** Definitions of normal ranges for cardiac MRI domain components. Ranges from Kawel-Boehm et al. (7).

**Measure**	**Normal range**
LV ejection fraction	51–79%
LV end diastolic volume	70–207 ml
RV ejection fraction	42–74%
RV end diastolic volume	68–244 ml
Left atrial length	4.2–7.1 cm
Left atrial width	3.1–5.3 cm
Right atrial length	4.0–6.6 cm
Right atrial width	3.2–5.9 cm
Septal thickness	0.5–1.2 cm
Lateral wall thickness	0.5–1.0 cm
Ascending aortic diameter	1.8–3.4 cm
Main pulmonary artery diameter	1.9–3.3 cm
LV mass	43–152 g
LV output	2.7–7.8 L/min
T1 time	885–1059 msec
T2 time	45–57 msec

LV, left ventricular; RV, right ventricular.

### Exposures and outcomes

Our primary exposure of interest was FIB-4 score within one year prior to CMR. FIB-4 is a non-invasive risk index associated with histological hepatic fibrosis, originally developed by Sterling et al. in HIV/Hepatitis C co-infected patients (8). It has been subsequently expanded to more general populations including NAFLD (9) and general primary care populations for screening purposes (10). We used the FIB-4 score, due to its applications for screening these more general populations, as well as the fact that it is composed of lab values derived from commonly acquired lab panels, thus decreasing selection bias. While multiple different cutoffs for FIB-4 exist, we categorized our population based off NAFLD screening cutoffs and a large community-based studies as low (< 1.3), moderate (1.3–3.25), and high (> 3.25) (11–14). If patients did not have each component lab value measured in a single visit, we calculated the FIB-4 score using relevant lab values within 24 hours of each other. For patients with multiple FIB-4 scores in the year prior to CMR, we computed the median FIB-4 score within the year.

Our primary outcome of interest was the presence of any abnormal result for any component of the CMR as described above. We also created binary variables to evaluate abnormalities across the four domains described above (compositional, functional, structural, vascular), and we defined the presence of any domain-specific abnormality as the occurrence of an abnormal value detected for any sub-component of each respective domain. For patients with multiple CMRs in a single year, we defined each outcome as the presence of abnormality across any CMR within that year.

### Statistical analysis

Demographic variables and clinical characteristics were expressed as a frequency (%) or mean (SD) as appropriate. Differences in characteristics across FIB-4 groups were assessed using ANOVA for continuous variables and chi-squared tests for categorical variables. We evaluated the association between FIB-4 score and domain-level CMR abnormalities using univariable and multivariable adjusted Poisson regression models with robust standard errors. Models were adjusted for demographics and traditional cardiac risk factors including age, sex, race, body mass index (BMI), hyperlipidemia, hypertension, diabetes, family history of premature coronary artery disease, and smoking. Among domains for which the association between FIB-4 score and domain-level abnormalities was statistically significant, we used univariable and multivariable Poisson regression models with robust standard errors to examine the association between FIB-4 score and sub-component abnormalities using the covariates described above. Additionally, we used further univariable and multivariable Poisson regression models with robust standard errors to examine whether associations between FIB-4 score and sub-component abnormalities were driven by abnormal values above vs. below the normal range. *P*-values below 0.05 were considered statistically significant. All analyses were performed using R statistical software version 4.0.3 (15).

## Results

We identified 1,668 patients who underwent CMR and had FIB-4 scores within the year prior to CMR. Among the study population, the mean FIB-4 score was 1.922 ± .70, with 794 (47.6%) were categorized as low FIB-4, 706 (42.3%) as moderate FIB-4, and 168 (10.1%) as high FIB-4 (Table 2). The mean age was 55.971 ± 7.28 years, with 54% male patients and 61.2% identifying as non-Hispanic White. Patients were on average not obese (mean BMI: 26.515 ± .50), but risk factors of hyperlipidemia (29.7%), diabetes (15.2%), and hypertension (39.2%) were relatively frequent. Differences between FIB-4 categories were seen for age (*p* < 0.001), sex (*p* < 0.001), hyperlipidemia (*p* < 0.001), family history of premature coronary artery disease (*p* = 0.024), diabetes (*p* < 0.001), and hypertension (*p* < 0.001).

**Table 2:** Demographic and clinical characteristics.

**Characteristics**	**Overall (*n* = 1,668)**	**Low FIB-4 (*n* = 794)**	**Moderate FIB-4 (*n* = 706)**	**High FIB-4 (*n* = 168)**	***P*-value**
**Demographic characteristics**					
Age, mean (SD)	55.97 (17.28)	46.76 (15.72)	64.38 (13.13)	64.20 (17.58)	<0.001
Male sex, *n* (%)	901 (54.0)	375 (47.2)	419 (59.3)	107 (63.7)	<0.001
Race, *n* (%)					0.82
Asian	115 (6.9)	53 (6.7)	51 (7.2)	11 (6.5)	
Hispanic/Latinx	199 (11.9)	103 (13.0)	74 (10.5)	22 (13.1)	
Non-Hispanic black	247 (14.8)	121 (15.2)	104 (14.7)	22 (13.1)	
Non-Hispanic white	1,021 (61.2)	476 (59.9)	442 (62.6)	103 (61.3)	
Other	72 (4.3)	37 (4.7)	27 (3.8)	8 (4.8)	
Unknown	14 (0.8)	4 (0.5)	8 (1.1)	2 (1.2)	
BMI, mean (SD)	26.5 (5.5)	26.7 (5.8)	26.4 (5.2)	26.0 (5.4)	0.27
**Risk factors**, ***n*** **(%)**					
Hyperlipidemia	495 (29.7)	197 (24.8)	266 (37.7)	32 (19.0)	<0.001
Family history of premature CAD	85 (5.1)	51 (6.4)	31 (4.4)	3 (1.8)	0.02
Diabetes	253 (15.2)	92 (11.6)	127 (18.0)	34 (20.2)	<0.001
Hypertension	654 (39.2)	249 (31.4)	341 (48.3)	64 (38.1)	<0.001
Smoking	38 (2.3)	12 (1.5)	22 (3.1)	4 (2.4)	0.12

CAD, coronary artery disease; SD, standard deviation.

Among the study population, 85.9% of patients had at least one cardiac or vascular abnormality detected in the CMR, with the prevalence of abnormalities increasing across low (82.0%) to moderate (88.8%) to high (92.3%) FIB-4 categories. Across domains, structural abnormalities were the most prevalent (65.0% overall; 66.2 vs. 67.8 vs. 66.7% for low, moderate, and high FIB-4 categories, respectively), followed by functional abnormalities (47.3%; 40.7 vs. 50.6 vs. 64.9%), compositional abnormalities (42.7%; 41.4 vs. 43.9 vs. 44.0%), and vascular abnormalities (28.8%; 20.3 vs. 36.4 vs. 36.9).

**Figure 1:**
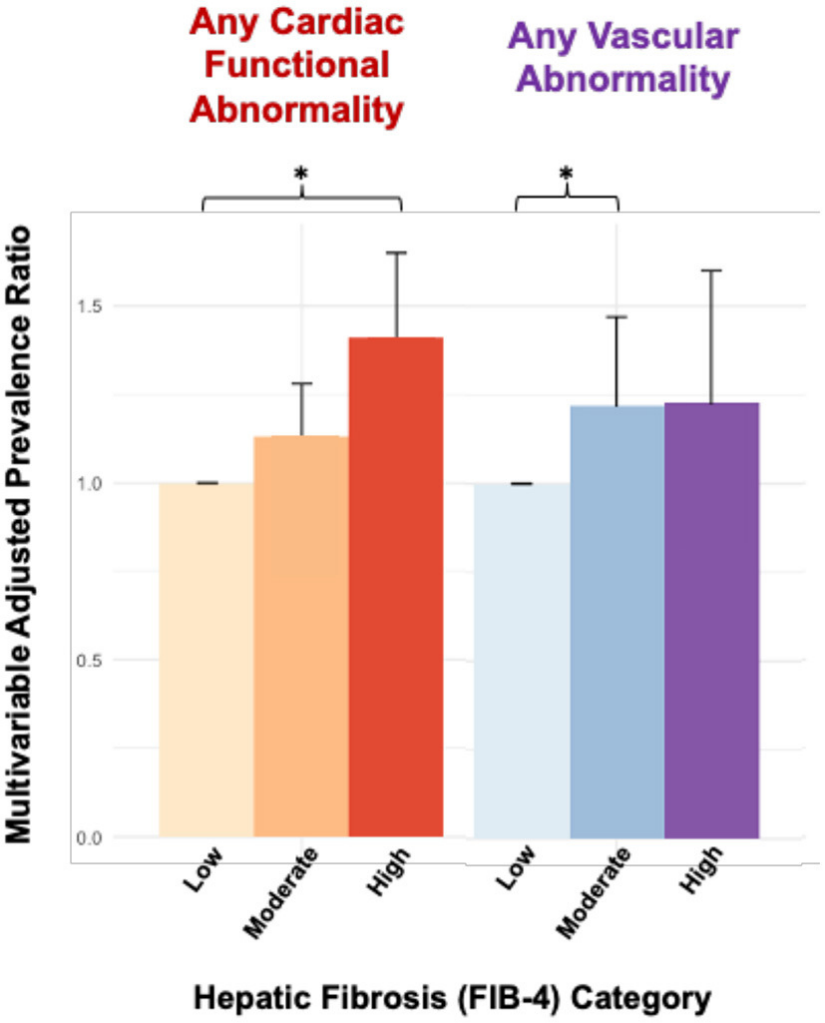
Cardiac MRI abnormalities associated with hepatic fibrosis by FIB-4. Bars are organized by FIB-4 score in ascending order (low, moderate, high) within each group. *p < 0.05, FIB-4 level low was used as reference.

In multivariable analyses, patients with high FIB-4 scores had a higher prevalence of any abnormality detected in the CMR when compared to patients with low FIB-4 scores (Prevalence Ratio (PR): 1.07, 95% CI:1.01–1.13), though no statistically significant differences were detected for patients in the moderate FIB-4 group (1.03, 0.98–1.08) (Table 3). In domain-level analyses, patients with high FIB-4 scores had a higher prevalence of functional abnormalities when compared to patients with low FIB-4 scores (1.41, 1.21–1.65), though no statistically significant differences were observed among the moderate FIB-4 group (1.13, 0.99–1.28). Patients with moderate FIB-4 scores had a higher prevalence of vascular abnormality when compared to patients with low FIB-4 scores (1.22, 1.01–1.47), though this difference was not observed among patients with high FIB-4 scores (1.23, 0.95–1.16), which may be related to sample size (Figure 1). No statistically significant differences were observed across FIB-4 groups for compositional or structural abnormality domains.

**Table 3:** Multivariable-adjusted prevalence ratio of cardiovascular abnormalities in association with hepatic fibrosis^*^.

	**Any cardiac or vascular abnormality**		**Any cardiac functional abnormality**		**Any vascular abnormality**	
**Liver FIB-4 score**	**Crude PR (95% CI)**	**Adjusted PR (95% CI)**	**Crude PR (95% CI)**	**Adjusted PR (95% CI)**	**Crude PR (95% CI)**	**Adjusted PR (95% CI)**
Low	Ref.	Ref.	Ref.	Ref.	Ref.	Ref.
Moderate	**1.08 (1.04, 1.13)**	1.03 (0.98, 1.08)	**1.24 (1.11, 1.39)**	1.13 (1.00, 1.28)	**1.80 (1.52, 2.13)**	**1.22 (1.01, 1.47)**
High	**1.13 (1.07, 1.19)**	**1.07 (1.01, 1.13)**	**1.59 (1.39, 1.83)**	**1.41 (1.21, 1.65)**	**1.82 (1.43, 2.32)**	1.23 (0.95, 1.6)
	**Any myocardial compositional abnormality**		**Any cardiac structural abnormality**	
**Liver FIB-4 score**	**Crude PR (95% CI)**	**Adjusted PR (95% CI)**	**Crude PR (95% CI)**	**Adjusted PR (95% CI)**
Low	Ref.	Ref.	Ref.	Ref.
Moderate	1.06 (0.94, 1.19)	1.02 (0.89, 1.17)	1.09 (1.01, 1.17)	1.00 (0.92, 1.08)
High	1.06 (0.88, 1.28)	1.01 (0.82, 1.24)	1.07 (0.95, 1.21)	0.95 (0.84, 1.08)

* Cardiac abnormalities were assessed by cardiac MRI, and hepatic fibrosis measure was based on the liver FIB-4 score.

Low FIB-4: < 1.3, Moderate FIB-4: ≥ 1.3 and < 3.25, High FIB-4 ≥ 3.25.

CI, confidence interval; PR, prevalence ratio.

Models adjusted for age, sex, race/ethnicity, BMI, hyperlipidemia, hypertension, diabetes, family history of coronary artery disease, and smoking status. Bold: *p* < 0.05.

In multivariable analyses assessing sub-components of domain-level abnormalities, domain-level findings appeared to be driven by abnormal left ventricular (LV) output and abnormal ascending aortic size in the function and vascular domains, respectively. When compared to patients with low FIB-4, patients with high FIB-4 had a higher prevalence of abnormal LV output (2.04, 1.31–3.17) (Table 4). No statistically significant differences were observed among wall motion abnormalities, LV ejection fraction, or right ventricular ejection fraction. Compared to patients with low FIB-4, patients with moderate or high FIB-4 had a higher prevalence of abnormal ascending aortic size (1.36, 1.07–1.72 and 1.39, 1.02–1.88 for moderate and high FIB-4, respectively). No statistically significant differences were observed for pulmonary arterial size abnormalities.

**Table 4:** Multivariable-adjusted prevalence ratio of cardiovascular abnormalities in association with hepatic fibrosis^*^, by sub-component.

**Liver FIB-4 score**	**Crude PR (95% CI)**	**Adjusted PR (95% CI)**	**Crude PR (95% CI)**	**Adjusted PR (95% CI)**	**Crude PR (95% CI)**	**Adjusted PR (95% CI)**
	**LV ejection fraction**		**LV output**		**Wall motion abnormalities**	
Low	Ref.	Ref.	Ref.	Ref.	Ref.	Ref.
Moderate	**1.37 (1.17, 1.59)**	1.11 (0.94, 1.32)	0.88 (0.63, 1.22)	1.07 (0.73, 1.57)	**1.31 (1.13, 1.51)**	1.09 (0.92, 1.28)
High	**1.51 (1.21, 1.88)**	1.18 (0.92, 1.52)	**1.75 (1.17, 2.63)**	**2.04 (1.31, 3.17)**	**1.58 (1.30, 1.94)**	1.25 (0.99, 1.58)
	**Ascending aortic size**		**Pulmonary arterial diameter**		**RV Ejection fraction**	
Low	Ref.	Ref.	Ref.	Ref.	Ref.	Ref.
Moderate	**2.33 (1.89, 2.87)**	**1.36 (1.07, 1.72)**	1.15 (0.83, 1.59)	1.27 (0.86, 1.88)	1 (0.79, 1.27)	0.95 (0.71, 1.26)
High	**2.62 (1.99, 3.44)**	**1.39 (1.02, 1.88)**	1.08 (0.63, 1.86)	1.17 (0.65, 2.12)	1.37 (0.99, 1.91)	1.27 (0.89, 1.83)

* Cardiac abnormalities were assessed by cardiac MRI, and hepatic fibrosis measure was based on the liver FIB-4 score.

Low FIB-4: < 1.3, Moderate FIB-4: ≥ 1.3 and < 3.25, High FIB-4 ≥ 3.25.

CI, confidence interval; CMR, cardiac MRI; LV, left ventricular; PR, prevalence ratio; RV, right ventricular.

Models adjusted for age, sex, race/ethnicity, BMI, hyperlipidemia, hypertension, diabetes, family history of coronary artery disease, and smoking status. Bold: *p* < 0.05.

Further analyses also revealed that findings for both LV output and ascending aortic size were driven primarily by values above, rather than below, the normal range. Among the *n* = 159 patients with abnormal LV output, 79% had LV output values above the normal range. In multivariable analyses, patients with high FIB-4 scores had a higher prevalence of elevated LV output compared to patients with low FIB-4 scores (2.74, 1.70–4.39) (Table 5), but no statistically significant difference was observed in the prevalences of depressed LV output. Similarly, among the n=378 patients with abnormal ascending aortic size, 99% had values above the normal range. In multivariable analyses, both moderate and high FIB-4 score patients had a greater prevalence of elevated ascending aortic size (1.38, 1.08–1.75 and 1.39, 1.02–1.89 for moderate and high, respectively). The accompanying model for low ascending aortic size was not run due to model convergence issues, as only *n* = 4 patients had ascending aorta size below the normal range.

**Table 5:** Multivariable-adjusted prevalence ratio of abnormal LV output and ascending aortic enlargement in hepatic fibrosis^*^.

	**High LV output**		**Low LV output**		**Enlarged ascending aortic size**	
**Liver FIB-4 score**	**Crude PR (95% CI)**	**Adjusted PR (95% CI)**	**Crude PR (95% CI)**	**Adjusted PR (95% CI)**	**Crude PR (95% CI)**	**Adjusted PR (95% CI)**
Low	Ref.	Ref.	Ref.	Ref.	Ref.	Ref.
Moderate	0.82 (0.56, 1.20)	1.09 (0.92, 1.28)	1.08 (0.54, 2.17)	1.11 (0.94, 1.32)	**2.42 (1.96, 3.00)**	0.95 (0.71, 1.26)
High	**1.98 (1.27, 3.08)**	1.25 (0.99, 1.58)	0.91 (0.27, 3.10)	1.18 (0.92, 1.52)	**2.72 (2.07, 3.59)**	1.27 (0.89, 1.83)

* Cardiac abnormalities were assessed by cardiac MRI, and hepatic fibrosis measure was based on the liver FIB-4 score.

CI, confidence interval; CMR, cardiac MRI; LV, left ventricular; PR, prevalence ratio; RV, right ventricular.

Low FIB-4: < 1.3, Moderate FIB-4: ≥ 1.3 and < 3.25, High FIB-4 ≥ 3.25.

Models adjusted for age, sex, race/ethnicity, BMI, hyperlipidemia, hypertension, diabetes, family history of coronary artery disease, and smoking status. Low ascending aortic size not evaluated due to model convergence issues (*n* = 4 patients with ascending aortic size below normal range). Bold: *p* < 0.05.

## Discussion

In our study, we assessed the association between FIB-4 score and abnormalities seen on CMR to elucidate the relationship between hepatic and cardiovascular disease. Our findings were twofold: first, high FIB-4 was associated with CMR functional and vascular abnormalities after multivariable adjustment for standard cardiovascular risk factors in a cohort of patients referred for CMR. Secondly, the abnormalities were driven by an increase in cardiac output in the high FIB-4 category and increased aortic size in both the moderate and high FIB-4 categories.

The associations between liver and cardiac disease using CMR have been limited thus far to small studies in cirrhosis and conditions such as NAFLD. Within cirrhosis, small studies have shown alterations in CMR measures of blood flow with elevated cardiac output and the increased presence of late gadolinium enhancement (16–18). A more recent study compared 42 patients with clinical cirrhosis undergoing evaluation for transhepatic portosystemic shunt insertion to 18 healthy control patients and found significant differences in function by longitudinal strain, and tissue characterization including T1 and T2 times, extracellular volume, and presence of LGE, with tissue-based abnormalities also being related to the severity of cirrhosis by Childs-Pugh classes (19). Within NAFLD, 19 adults with the condition were matched to healthy controls, with evidence of mildly increased wall-thickening, decreased longitudinal shortening, and increased strain without changes in cardiac metabolism measured by 31P-Magnetic resonance spectroscopy (20). Our results appear to be different from some of these results, which may be a function of our design. Specifically, our population was not separated into more distinct groups of clinically advanced-stage cirrhotic patients compared to healthy controls, which may affect our ability to see subtle changes in wall thickness or tissue characteristics. Additionally, in prior studies, matching for cardiovascular risk factors was either severely limited (e.g. age, sex, BMI) or not performed at all. Therefore, differences in the presence of LGE may not be evident due to this being a fully clinically-referred population representing potentially higher cardiovascular risk, in which the presence of LGE due to other etiologies may be more common despite adjustment for cardiovascular risk factors. Our sample size made it less feasible to perform individual strain analyses on the patients; therefore, we lacked the ability to confirm certain differences. Finally, in order to maintain statistical validity with multiple testing, given the plethora of measurements that a single CMR provides, we attempted to categorize and dichotomize the presence vs. absence of abnormality, which likely results in limited comparison for groups who may have differences but are within the range of normal.

Our results are in line with previous results but cover a broader spectrum of potentially subclinical diseases. In general, we observed a stepwise increase in the prevalence of abnormalities across the increasing FIB-4 groups, which gives biological plausibility as a dose response for the abnormalities. Within the multivariable analysis, the increase in cardiac output abnormalities is likely related to the distributive/high-flow state seen in more advanced cirrhosis (21). This is supported, with only the high FIB-4 category having significant abnormalities and the intermediate category being non-significant. It is uncertain in our study whether or not the increases in cardiac output are driven by heart rate. stroke volume, or other etiologies, though decreased total peripheral resistance and increased stroke volume have been previously noted in cirrhosis (22, 23). More interestingly, the differences in ascending aortic diameter were seen in both moderate and high FIB-4 categories, despite adjustment for body size, sex, and cardiovascular risk factors. This may suggest a component of vascular disease, dilation, or dysfunction seen in subclinical hepatic disease states or at least preceding overt advanced hepatic fibrosis. In particular, vasodilation as a pre-high-flow state may explain these abnormalities; however, whether this vascular disease is due to inflammation, endothelial dysfunction, pro-fibrotic states, accelerated atherosclerosis, or other etiologies is unknown. All of these pathways have been potentially implicated as dysfunctional within NAFLD patients (24). Further investigations of vascular abnormalities in the context of both subclinical and clinical hepatic fibrosis are warranted.

Our study has some limitations that merit discussion. As a retrospective cohort study of patients clinically referred for CMR, our analysis was focused on individuals who are likely to have a higher prevalence of abnormalities than in an unselected community-based population; thus, while broad generalizability may be limited, the findings are potentially more relevant to individuals encountered in the clinical care setting and more amenable to future interventions that medical providers could facilitate. Similarly, restriction of analyses to patients with available laboratory values sufficient to calculate a FIB-4 may sub-select a cohort more engaged with the medical system due to comorbidities. Finally, although FIB-4 has been validated as a method for screening for hepatic fibrosis, it is not as sensitive or specific compared to the gold standard liver biopsy-based assessments or more specific imaging or serological methods, its individual components (i.e. platelet counts and transaminases) may be affected by acute hepatic injury or non-hepatic conditions which may increase noise within our models, and specific cutoffs may have variable accuracy between different disease or demographic populations. Notwithstanding the limitations, a strength of our study includes the use of validated measures and the inclusion of a much larger cohort than those included in prior reports. Nonetheless, given our study's cross-sectional design, we consider our findings as hypothesis-generating with respect to putative causality or directionality of heart-liver axis associations. Additional studies in separate and more diverse populations using more specific measures of liver fibrosis are needed to validate our findings.

In conclusion, we found in a large cohort of real-world patients referred for CMR evaluated in a large healthcare setting that the presence of cardiovascular abnormalities was significantly associated with measures of hepatic fibrosis. These abnormalities were most notable for alterations in cardiac function, driven by cardiac output, and alterations in vascular anatomy, driven by ascending aortic size. These cardiovascular findings appeared related to hepatic fibrosis, even after accounting for the presence of shared risk factors—underscoring the importance of further consideration of and investigations into perturbations of the heart-liver axis and their longer-term clinical implications for affected individuals.

## Data availability statement

The raw data supporting the conclusions of this article will be made available by the authors, without undue reservation.

## Ethics statement

This study involving human participants was reviewed and approved by Cedars Sinai IRB. Written informed consent for participation was not required for this study due to retrospective nature in accordance with the national legislation and the institutional requirements.

## Author contributions

AK was involved in conceptualization, formal analysis, investigation, writing—original draft, writing—review and editing, and visualization. NS and MD were involved in methodology, formal analysis, investigation, and visualization. PB and JN were involved in methodology, formal analysis, and data curation. DO was involved in methodology, investigation, and writing—review and editing. SH was involved in conceptualization, methodology, and writing—review and editing. MN was involved in conceptualization, methodology, resources, writing—review and editing, and supervision. DL was involved in resources, writing—review and editing, and supervision. JE was involved in methodology, resources, data curation, and writing—review and editing. DB was involved in resources, data curation, writing—review and editing, and supervision. SC was involved in conceptualization, methodology, resources, writing—original draft, writing—review and editing, visualization, and supervision. All authors contributed to the article and approved the submitted version.

## Funding

AK reports funding support from the Doris Duke Charitable Foundation Grant 2020059, a Smidt Heart Institute Research Grant, and National Institutes of Health 2R01HL131532-06A1. DO reports funding support from National Institutes of Health K99HL157421-01. JE reports funding support from National Institutes of Health K23HL153888.

## Conflict of interest

The authors declare that the research was conducted in the absence of any commercial or financial relationships that could be construed as a potential conflict of interest.

## Publisher's note

All claims expressed in this article are solely those of the authors and do not necessarily represent those of their affiliated organizations, or those of the publisher, the editors and the reviewers. Any product that may be evaluated in this article, or claim that may be made by its manufacturer, is not guaranteed or endorsed by the publisher.

## Abbreviations

BMI: Body Mass Index
CMR: Cardiac MRI
EMR: Electronic Medical Record
HASTE: Half-Fourier Acquisition Single-shot Turbo spin Echo
LGE: Late Gadolinium Enhancement
LV: Left ventricle
NAFLD: Non-alcoholic Fatty Liver Disease
SSFP: Steady-state free precession.
